# Bibliometric Analysis and Visualization of Research Progress in the Diabetic Nephropathy Field from 2001 to 2021

**DOI:** 10.1155/2023/4555609

**Published:** 2023-01-21

**Authors:** Ying Shao, Xiaoguang Shi

**Affiliations:** Department of Endocrinology, Shengjing Hospital of China Medical University, Shenyang, Liaoning, China

## Abstract

**Methods:**

The PubMed database was searched to identify all studies related to DN that were published from 2001 to 2021, with these studies being separated into four time-based groups. The characteristics of these studies were analyzed and extracted using BICOMB. Biclustering analyses for each of these groups were then performed using gCLUTO, with these results then being analyzed and GraphPad Prism 5 being used to construct strategy diagrams. The social network analyses (SNAs) for each group of studies were conducted using NetDraw and UCINET.

**Results:**

In total, 18,889 DN-associated studies published from 2001 to 2021 and included in the PubMed database were incorporated into the present bibliometric analysis. Biclustering analysis and strategy diagrams revealed that active areas of research interest in the DN field include studies of the drug-based treatment, diagnosis, etiology, pathology, physiopathology, and epidemiology of DN. The specific research topics associated with these individual areas, however, have evolved over time in a dynamic manner. Strategy diagrams and SNA results revealed podocyte metabolism as an emerging research hotspot in the DN research field from 2010 to 2015, while DN-related microRNAs, signal transduction, and mesangial cell metabolism have emerged as more recent research hotspots in the interval from 2016 to 2021.

**Conclusion:**

Through analyses of PubMed-indexed studies pertaining to DN published since 2001, the results of this bibliometric analysis offer a knowledge framework and insight into active and historical research hotspots in the DN research space, enabling investigators to readily understand the dynamic evolution of this field over the past two decades. Importantly, these analyses also enable the prediction of future DN-related research hotspots, thereby potentially guiding more focused and impactful research efforts.

## 1. Introduction

The incidence of metabolic diseases has been steadily rising throughout the world in the 21^st^ century, with rates of type 2 diabetes mellitus (T2DM) being particularly high [1]. Diabetic nephropathy (DN) is a microvascular complication that often develops in diabetic patients and is currently the leading global cause of chronic kidney disease, imposing a significant burden on patients [2]. As the primary cause of end-stage renal disease (ESRD), diabetes mellitus (DM) is also contributing to higher rates of patient mortality [3]. As such, DN has emerged as a key focus of diabetes-related research interest, with large quantities of complex studies focused on DN having been published over the past two decades. The specific hotspots of interest within this DN field have dynamically evolved over time, changing based on prevailing research trends and important breakthroughs. Many of these studies have conducted detailed analyses exploring specific topics such as the most effective approaches to treating DN [4]; the pathology, etiology [5], or epidemiology of this condition [6]; biomarkers and other factors associated with DN diagnosis [7]; and other details pertaining to DN pathogenesis [8]. These studies have produced a wealth of data, yet each has a distinct focus. Given the complexities of these studies and the intricately detailed relationships among DN-related research hotspots, there is a critical need for a systematic and scientific exploration of the knowledge framework in this field so as to guide future research efforts. In this case, scientific research clearly requires cooperative efforts. And bibliometric analysis is one of the cooperative methods [9].

The science of bibliometrics [10] allows researchers to gain objective and comprehensive insights into the development and characteristics of a given research discipline via quantitatively analyzing the corresponding literature base. These bibliometric analyses can additionally provide a means of objectively gauging the influence of particular key publications in a given field [11]. Recent advances in computer-based technologies have led to the emergence of several bibliometric analysis programs including the Bibliographic Item Co-Occurrence Matrix Builder (BICOMB), UCINET, gCLUTO, and NetDraw. Of these, BICOMB enables co-word analyses that can rapidly extract critical information from thousands of manuscripts, while gCLUTO can leverage the results of these co-word analyses to perform result biclustering and visualization. These data can then be further analyzed through the construction of strategy diagrams and SNA graphs that offer insights into the corresponding knowledge framework and dynamic evolution of a field, thereby enabling the prediction of future research hotspots. These programs are highly accessible, easily enabling researchers and other professionals to visualize and interpret the resultant data.

For the present analysis, the 21 years were separated into four time periods (2001-2005, 2006-2010, 2011-2015, and 2016-2021) to more effectively monitor research trends. Bibliometric analyses were then employed to explore DN-related studies published in the PubMed database during these time periods in order to better define the corresponding knowledge framework and to generate corresponding visualizations. Since this is a medical subject, the data found in PubMed might be more helpful than those found in Web of Science (WOS). The overarching goal of this analysis is to provide researchers with insights into the hotspots of active research interest in the DN field and how these have changed over time in order to better understand the development of this research discipline. In addition, an objective approach is used to identify potential future DN-related research hotspots so as to inform ongoing scientific efforts.

## 2. Materials and Methods

### 2.1. Data Collection

For this bibliometric analysis, all articles were retrieved from the PubMed database using Medical Subject Headings- (MeSH-) indexing terms as a means of effectively identifying relevant studies of interest. For this literature search, no language, literature type, or species restrictions were imposed in order to obtain the maximum possible amount of DN-related information. “Diabetic nephropathies” was selected as the MeSH search term. To monitor the development of this research field, publications were separated into four time periods: 1 January 2001 to 31 December 2005, 1 January 2006 to 31 December 2010, 1 January 2011 to 31 December 2015, and 1 January 2016 to 31 December 2021. To avoid potential bias as a consequence of updates to the PubMed database, the retrieval and downloading of 18,889 publications associated with these four time periods were completed on 2 January 2022, with two authors having independently verified that this was completed successfully.

### 2.2. Data Extraction and Matrix Generation

Key features were extracted from retrieved publications using the BICOMB [12], which extracted MeSH term/subheading, journal, and country of publication for all four time intervals. Terms-publication and co-occurrence matrices were then generated [13]. The H-index is a commonly used metric for the value of scientific research [14, 15], and MeSH term/subheading word frequency orders were thus sorted from high to low for each time interval such that the H-index method could be employed to effectively identify those high-frequency MeSH terms/subheadings for each time period [16].

### 2.3. Biclustering Analyses

The four term-publication matrices generated with the BICOMB program consisting of PubMed Unique Identifiers (PMIDs) and high-frequency MeSH terms/subheadings were next analyzed using gCLUTO [17]. Biclustering results were used to construct four heat maps, with columns and rows corresponding to PMIDs and high-frequency MeSH terms/subheadings, respectively. Dendrograms on the upper and left of the heat map were additionally included. These results were further used to generate three-dimensional (3D) mountain diagrams in which the peak volume correlates with numbers of high-frequency MeSH terms/subheadings in a given cluster, while the peak height is proportional to within-cluster similarity, and the distance between peaks is directly proportional to the similarity within the cluster. The color of the apex of each peak was based on the intracluster variation, with red and blue corresponding, respectively, to a small and large degree of variation. Each cluster from the four time periods of interest was then analyzed and summarized based on biclustering analysis results.

### 2.4. Strategy Diagram Analyses

To better understand the knowledge framework and dynamic evolution of the DN field over the past two decades, four two-dimensional (2D) strategy diagrams were constructed. These diagrams offer a visual means of describing external and internal connections for a particular research field [18, 19], with *X* and *Y* coordinates serving as the most critical indicators for summarizing these diagrams. Coordinate calculation methods have been described in prior reports [20]. In the resultant diagrams, centrality, which reflects the strength of the relationships between one topic and other topics, is represented by the *x*-axis, while density values, which correspond to topic interconnectedness (as a measure of topic maturity), are represented by the *y*-axis. The co-occurrence matrices constructed for each time period were used to compute these coordinate values, after which corresponding strategy diagrams were constructed with GraphPad Prism 5.

### 2.5. Social Network Analyses (SNAs)

SNAs are statistical data-mining strategies that enable the exploration of the internal structures and relationships for particular groups in the form of a visual network structure diagram [21, 22]. The key metric used to describe these SNA graphs is centrality, which includes degree, betweenness, and closeness centrality. Of these, degree centrality served as the most direct metric for the value of a given node within the SNA network, while betweenness centrality corresponds to the number of shortest paths through the node and thus reflects node importance. Closeness centrality represents the reciprocal of the sum of the shortest paths from a node to all reachable nodes and thus reflects the proximity of a given node to all other nodes in that network [23].

For the present study, BICOMB was used to construct high-frequency MeSH term/subheading co-occurrence matrices for four time periods, while these three centrality values were calculated for each period using UCINET. SNA visualization was then performed using NetDraw, with betweenness centrality being represented by node size such that the largest node in the network is the hub node. Line thickness, in contrast, corresponds to the frequency of cooccurrence for high-frequency MeSH terms/subheadings.

## 3. Results

### 3.1. Characteristics of DN-Related Studies Published from 2001 to 2021

**Figure 1:**
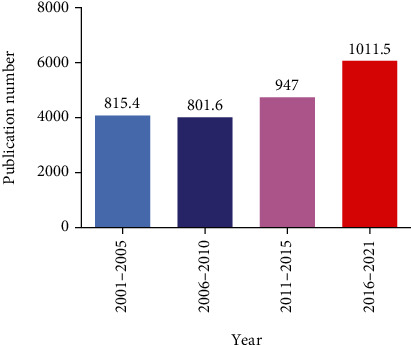
Numbers of DN-related studies published from 2001 to 2021.

Through the search strategy detailed above, 4077, 4008, 4735, and 6069 DN-related studies were identified for the intervals from 2001 to 2005, 2006 to 2010, 2011 to 2015, and 2016 to 2021, respectively, with average annual publication numbers during these four respective periods of 815.4, 801.6, 947, and 1011.5 (Figure 1). While the number of publications declined slightly from 2006 to 2010, the overall number of DN-related publications has continued to rise over the course of the 21^st^ century with some degree of fluctuation. The top 10 countries and journals associated with research output in the DN field over this two-decade period are summarized in Table 1. While the United States was the country with the largest DN-related publication output during these four periods, its overall proportional contribution to this field has continued to decline. In contrast, the United Kingdom exhibited the second highest publication output over this period, and its proportional output has continued to rise. Germany was the third most productive country during the first three analyzed time periods, although the Netherlands ranked third during the interval from 2016 to 2021.

The journals associated with the greatest number of DN-related publications during the first two analyzed time periods were *Kidney International*, *Diabetes Care*, and *Nephrol Dial Transplant*, accounting for 13.8% and 11.5% of the total DN-related publications from 2001 to 2005 and 2006 to 2010, respectively. While *Kidney International* remained the second most prolific journal from 2011 to 2015 in this research space, *PLOS ONE* and the *Journal of Diabetes and Its Complications*, respectively, ranked first and third. The top three most productive DN-related journals from 2016 to 2021 were *Scientific Reports*, *Journal of Diabetes Research*, and the *Journal of Diabetes and Its Complications*.

### 3.2. Biclustering Analysis of High-Frequency MeSH Terms/Subheadings in the DN Research Field

**Figure 2:**
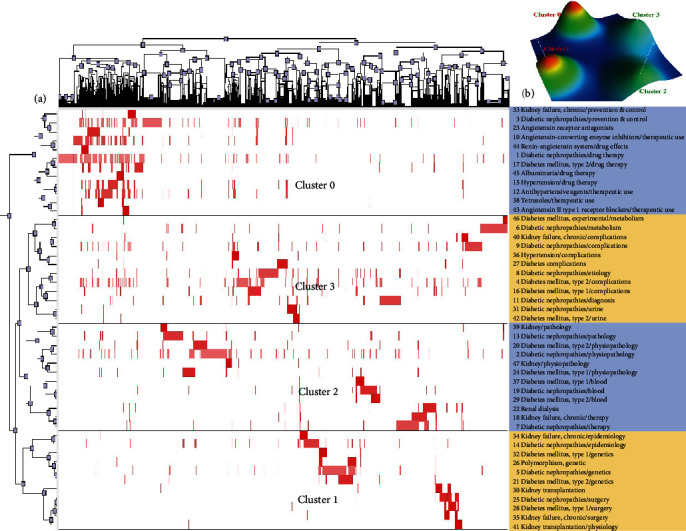
Biclustering analysis corresponding to 47 high-frequency MeSH terms/subheadings associated with DN-related publications from 2001 to 2005. (a) Matrix visualization of the results of the biclustering analysis of 47 high-frequency MeSH terms/subheadings and corresponding publication PMIDs. (b) Mountain visualization of the results of this biclustering analysis.

In total, the 47, 49, 49, and 59 highest frequency MeSH terms/subheadings were extracted from 4077, 4008, 4735, and 6069 DN-related publications, respectively, over the corresponding time periods, with respective cumulative frequency values of 35.85%, 33.79%, 34.49%, and 38.89%. These high-frequency MeSH terms/subheadings represent hotspots of DN-related research interest over the past two decades. The 48 highest frequency MeSH terms/subheadings from the 2001-2005 period were next clustered into four clusters via a biclustering analysis approach (Figure 2), with these clusters additionally being represented with a mountain diagram (Figure 2(b)). The highest frequency MeSH terms/subheadings associated with each cluster are shown in the column to the right in Figure 2(a), showing clustering relationships between high-frequency MeSH terms/subheadings and the corresponding literature.

**Figure 3:**
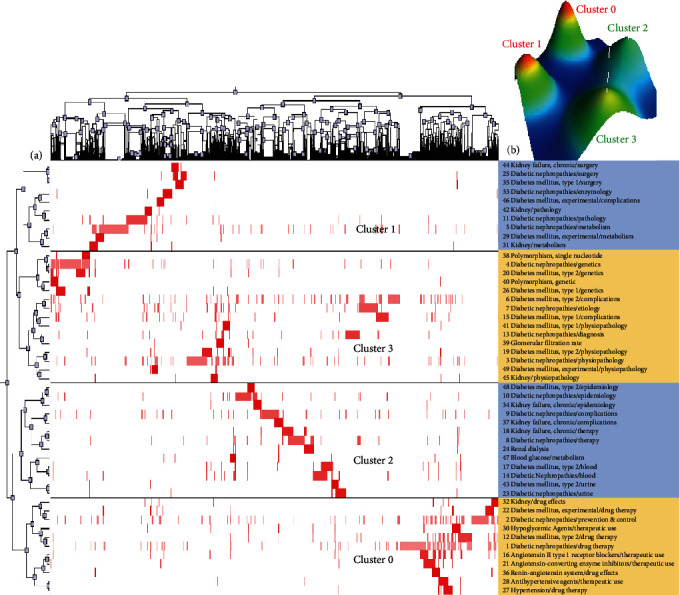
Biclustering analysis corresponding to 49 high-frequency MeSH terms/subheadings associated with DN-related publications from 2006 to 2010. (a) Matrix visualization of the results of the biclustering analysis of 49 high-frequency MeSH terms/subheadings and corresponding publication PMIDs. (b) Mountain visualization of the results of this biclustering analysis.

**Figure 4:**
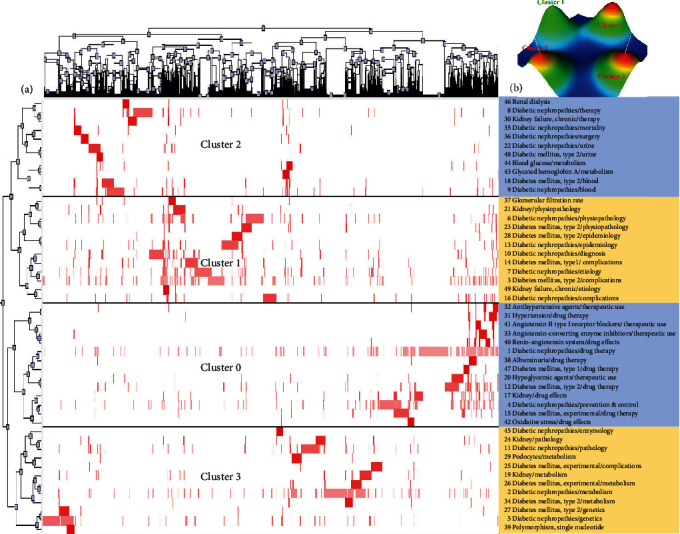
Biclustering analysis corresponding to 49 high-frequency MeSH terms/subheadings associated with DN-related publications from 2011 to 2015. (a) Matrix visualization of the results of the biclustering analysis of 49 high-frequency MeSH terms/subheadings and corresponding publication PMIDs. (b) Mountain visualization of the results of this biclustering analysis.

**Figure 5:**
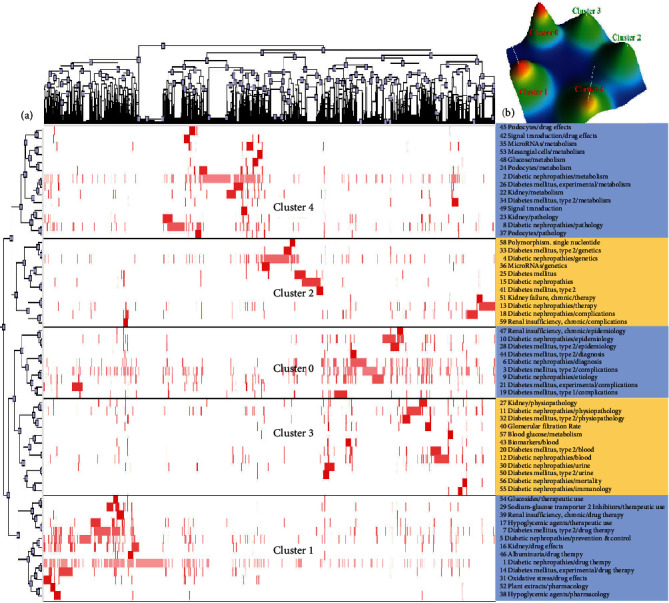
Biclustering analysis corresponding to 59 high-frequency MeSH terms/subheadings associated with DN-related publications from 2016 to 2021. (a) Matrix visualization of the results of the biclustering analysis of 59 high-frequency MeSH terms/subheadings and corresponding publication PMIDs. (b) Mountain visualization of the results of this biclustering analysis.

The 49, 49, and 59 high-frequency MeSH terms/subheadings extracted for the 2006-2010, 2011-2015, and 2016-2021 intervals were similarly grouped by biclustering analyses into 4, 4, and 5 respective clusters as shown in Figures 3–5, with the results of analyses of these clusters being compiled in Table 2.

### 3.3. Strategic Diagram Analyses of the DN Research Space

**Figure 6:**
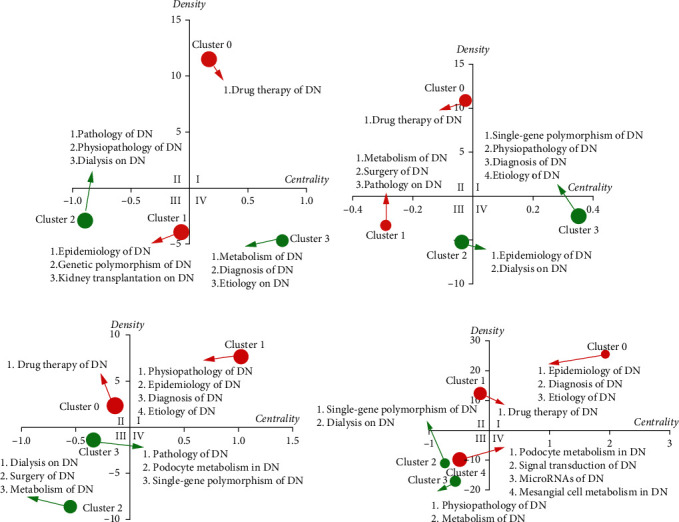
Strategic diagram analyses of the DN field. Strategic diagrams were constructed corresponding to DN-related research during the periods from (a) 2001 to 2005, (b) 2006 to 2010, (c) 2011 to 2015, and (d) 2016 to 2021. Clusters in each of these diagrams correspond to the biclustering results shown in Table 2. Signal node sizes correspond to numbers of high-frequency MeSH terms/subheadings in that cluster.

Next, strategic diagrams were constructed to summarize this DN-related research space, with the *x*-axis of these diagrams corresponding to degree centrality as a matrix of the core degree of a given cluster in a particular research space, while the *y*-axis corresponds to density and reflects the internal characteristics of that cluster. Higher density values correspond to a closer relationship within that cluster consistent with greater cluster maturation. When the resultant diagram is separated into four sections, quadrant I (upper right) corresponds to topics that exhibit high degrees of both centrality and maturity, while those in quadrant II (upper left) correspond to mature but less central research topics. Topics within quadrant III (lower left) exhibit lower centrality and density values and, thus, may correspond to newly emergent or declining directions of research interest. Notably, those topic clusters included within quadrant IV (lower right) exhibit a high degree of centrality but low maturity and may thus correspond to emerging research topics in which there is a high degree of interest. Node size in these strategy diagrams is proportional to the numbers of high-frequency MeSH terms/subheadings in a given cluster (Figure 6), with the four resultant diagrams thus corresponding to trends in the DN field over the four analyzed periods during the 21^st^ century. Comparisons of these data can be used to gain insight into the dynamic evolution of this research space.

From 2001 to 2005, Cluster 0 (drug therapy of DN) was located within quadrant I, indicating that this was a highly developed topic of focused research interest with high density and centrality values. During this same interval, Clusters 1 and 2, which represented DN-related genetic polymorphisms, epidemiology, pathology, pathophysiology, dialysis, and kidney transplantation, were located in quadrant III, indicating that these were still relatively immature topics of research interest at the fringes of the field during this time. Cluster 3, which corresponded to the diagnosis, etiology, and metabolism of DN, was located in quadrant IV during this period, indicating that while these topics were associated with a high core degree value, they were still relatively immature research areas.

From 2005 to 2010, Cluster 0 (drug therapy of DN) moved to quadrant II, suggesting the weakening of the core position of this mature research area within the overall field. Moreover, Clusters 1 and 2, corresponding to the DN-related metabolism, surgery, pathology, epidemiology, and dialysis publications, were located in quadrant III, indicating that these research directions remained fairly immature and at the fringes of the overall field during this period. Cluster 3, corresponding to DN-related single-gene polymorphisms, the pathophysiology of DN, and the diagnosis and etiology of DN, was located in quadrant IV, highlighting these as increasingly central research topics within the DN field that were nonetheless relatively immature at this point in time.

During the interval from 2011 to 2015, the cluster corresponding to the pathophysiology, etiology, diagnosis, and epidemiology of DN moved to quadrant I consistent with the further development and maturation of these research topics. As during the previous 5-year period, Cluster 0 (drug therapy of DN) remained in quadrant II, while quadrant III contained Cluster 2 (DN-related dialysis, surgery, and metabolism research) and Cluster 3 (DN-related pathology, single-gene polymorphism, and podocyte metabolism research). As such, these topics of research interest remained at the edges of the DN field during this time period and were relatively immature, although they have the potential to include certain research hotspots.

Over the period from 2016 to 2021, the epidemiology, diagnosis, and etiology of DN remained in quadrant I corresponding to mature topics of core research interest in this field, while drug therapy of DN remained in quadrant III. The pathophysiology of DN, which was located in quadrant I during the previous time interval, had relocated to quadrant III, while DN-related microRNA (miRNA), signal transduction, and mesangial cell metabolism research were newly located within this quadrant, suggesting these to be emerging directions of research interest in the DN field.

### 3.4. Social Network Analysis

**Figure 7:**
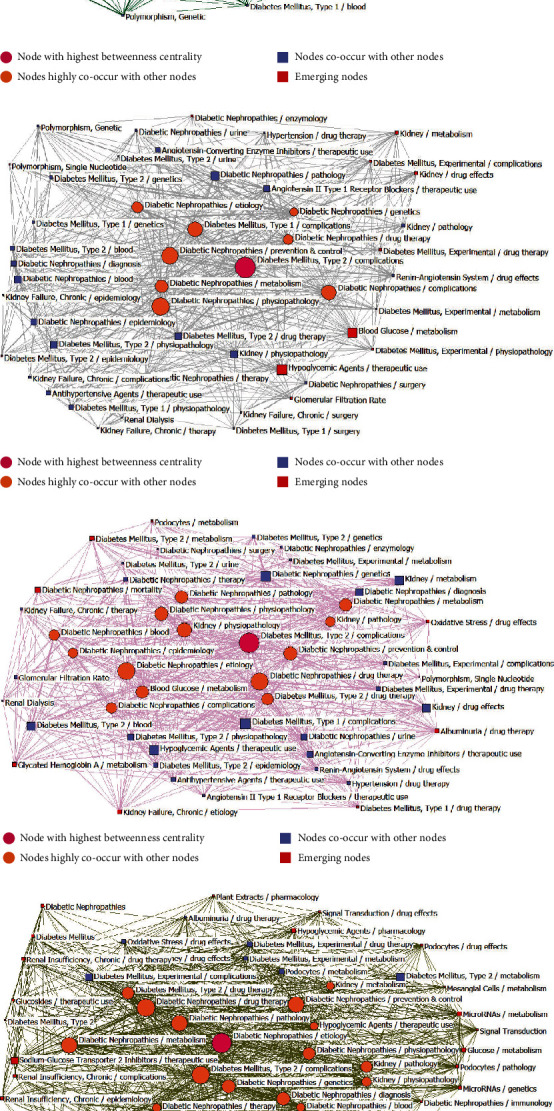
Social network analyses (SNAs) of high-frequency MeSH terms/subheadings associated with DN-related publications from 2001 to 2021. (a) SNA of 47 high-frequency MeSH terms/subheadings from 2001 to 2005. (b) SNA of 49 high-frequency MeSH terms/subheadings from 2006 to 2010. (c) SNA of 49 high-frequency MeSH terms/subheadings from 2011 to 2015. (d) SNA of 59 high-frequency MeSH terms/subheadings from 2016 to 2021.

Next, four SNA diagrams were constructed to explore the structure of the DN-related research space through the calculation of the degree centrality, betweenness centrality, and closeness centrality parameters (Figure 7). As betweenness centrality is particularly important, these diagrams were primarily based upon this parameter, with the node size being proportional to the betweenness centrality for the included high-frequency MeSH terms/subheadings, while the line thickness corresponds to the co-occurrence frequency.

For the SNA diagram from 2001 to 2005, 8 MeSH terms/subheadings were found to exhibit a high level of betweenness centrality, with the two exhibiting the highest such centrality being Diabetes Mellitus Type 2/complications (57.78123) and Diabetic Nephropathies/Prevention and Control (53.94703). These two terms are thus pivotal to the network as a whole. The 6 other terms with the highest level of betweenness centrality were Diabetes Mellitus Type 1/complications, Diabetic Nephropathies/complications, Renal Dialysis Diabetic Nephropathies/epidemiology, Diabetic Nephropathies/etiology, and Diabetic Nephropathies/drug therapy. Nodes corresponding to these terms thus served as bridges within the overall network. The average betweenness centrality value for the overall SNA network during this time interval was 11.702 ± 12.722. The two terms exhibiting the highest degree centrality in this network were also among these top 8 MeSH terms/subheadings, including Diabetic Nephropathies/drug therapy (606) and Diabetes Mellitus Type 2/complications (511). These two terms/subheadings thus occupy a core position within the constructed network, which had an overall average degree centrality of 181.277 ± 133.242. The nodes with the highest closeness centrality were Diabetes Mellitus Experimental/metabolism (88), Diabetes Mellitus Type 1/surgery (87), and Kidney Transplantation/Physiology (86). As they were located on the periphery of this SNA network, this suggests that they may represent emerging high-frequency MeSH terms/subheadings during this period. Overall, this network had an average closeness centrality of 69.404 ± 9.11.

Relative to the period from 2001 to 2005, 11 additional high-frequency MeSH terms/subheadings were incorporated into the SNA from 2006 to 2010. These included Hypoglycemic Agents/therapeutic use (23.922) and Blood Glucose/metabolism (20.785), which exhibited the highest betweenness centrality values. The other 9 new MeSH terms/subheadings included Polymorphism and Single Nucleotide and were located on the periphery of the overall SNA network, suggesting them to be potentially emerging hotspots of scientific interest over this time frame. The average degree centrality, betweenness centrality, and closeness centrality values for this SNA network were 11.735 ± 12.889, 152.816 ± 100.376, and 71.469 ± 9.2873, respectively (Figure 7(b)).

Relative to the 2006-2010 period, 8 additional high-frequency MeSH terms/subheadings were incorporated into the SNA network for the period from 2011 to 2015, including Podocytes/metabolism and Oxidative Stress/drug effects. These terms were located in different regions of the network and correspond to emerging research hotspots over this interval (Figure 7(c)). The average respective degree centrality, betweenness centrality, and closeness centrality values for this SNA network were 9.388 ± 7.731, 237.714 ± 174.789, and 66.776 ± 8.630, respectively.

Relative to the 2011-2015 period, 21 additional high-frequency MeSH terms/subheadings were incorporated into the SNA for the 2016-2021 period, with the three MeSH terms/subheadings exhibiting the highest level of betweenness centrality being Sodium-glucose Transporter 2 Inhibitors/therapeutic use (13.413), MicroRNAs/metabolism (7.425), and Hypoglycemic Agents/Pharmacology (6.430). Despite being newly emergent research foci within this field, they nonetheless played an important mediatory role within the SNA network over this time period. Other newly emergent MeSH terms/subheadings included Plant Extracts/pharmacology, Mesangial Cells/metabolism, MicroRNAs/genetics, Podocytes/pathology, Biomarkers/blood, Podocytes/drug effects, Glucose/metabolism, Signal Transduction/drug effects, Glucosides/therapeutic use, and Diabetic Nephropathies/immunology. These terms may also correspond to emerging directions for intensive research interest over this interval. The average respective degree centrality, betweenness centrality, and closeness centrality values for this network were 12.305 ± 11.148, 313.356 ± 283.332, and 82.610 ± 10.386, respectively (Figure 7(d)).

## 4. Discussion

There have been over 18,000 DN-related manuscripts published since the beginning of the 21^st^ century corresponding to a diverse array of topics including DN-related drug therapy [24–26], the pathology and pathophysiology of DN [27], the diagnosis of DN [28, 29], the etiology of DN [30, 31], and the epidemiology of DN [32, 33]. Given the complexity and density of information published in this field over the past two decades, there is a clear need for a systematic and objective approach to surveying DN-related research directions. As such, the present bibliometric analysis was conducted, employing co-word, biclustering, strategy diagram, and SNA metrology approaches to visualize the DN-related knowledge framework since 2001 and to forecast future hotspots for intensive research interest. Through these approaches, this study offers insight regarding the dynamic evolution of DN-related research over the past 20 years and the likely directions for this field in the near future.

For this analysis, the past 21 years were separated into four periods (2001-2005, 2006-2010, 2011-2015, and 2016-2021). Over this interval, the number of DN-related publications tended to increase with some fluctuations, although the overall research output in this space remained high, suggesting this to be a key area of sustained diabetes- and metabolism-related research interest.

Strategy diagram results revealed the drug therapy of DN to be located in quadrant I from 2001 to 2005 but in quadrant II from 2006 to 2021. This indicates that this research area has remained mature owing to a high degree of sustained research interest over the past two decades but that its core status within the overall DN field has declined somewhat with time. An in-depth characterization of studies associated with this topic revealed that renin-angiotensin-aldosterone system (RAAS) blockage and antihypertensive drugs formed the core of drug-based approaches to DN treatment from 2001 to 2005. During this period, the foundation for utilizing RAAS blockers to treat DN was established, with both angiotensin receptor blocker (ARBs) and angiotensin-converting-enzyme inhibitor (ACEIs) drugs effectively protecting against DN through RAAS-blocking activity, leading to improvements in proteinuria, renal blood flow, and blood pressure [34]. Biclustering analysis results suggested that further progress in the drug-based treatment of DN was made from 2006 to 2010, with researchers having demonstrated the protective benefits of hypoglycemic drugs in the treatment of DN, in line with the results of corresponding SNAs. During this period, studies established the ability of advanced glycation end products (AGEs) to drive the pathogenesis of DN through oxidative stress and inflammatory signaling, with hypoglycemic drugs preventing renal AGE accumulation and thus improving kidney function [35]. SNAs indicated that from 2011 to 2015, the antioxidative stress drug-based treatment of DN emerged as a new research hotspot, in line with biclustering analysis results. During the early portion of this period, some studies found a putative link between the excessive oxidative stress in circulation and that in the cellular microenvironment that contributed to dysfunctional endothelial cell activity, in turn contributing to DN onset and progression. As few effective antioxidant drugs were available at this time, however, it was primarily suggested that lifestyle interventions including the cessation of smoking and increased exercise be used to improve patient outcomes pending the development of more effective pharmacological tools [36]. During the later time points of this period, a series of antioxidant drugs were developed that were found to improve DN. Moreover, Wakino et al. [37] identified a protective role for Sirtuin 1 (Sirt1) in DN associated with antioxidant stress. During the period from 2016 to 2021, the results of both biclustering analyses and SNAs indicated that plant extract-based drugs and sodium-glucose cotransporter-2 (SGLT2) inhibitors have emerged as promising directions for the drug-based treatment of DN. The Polygonaceae-derived *Polygonum aviculare L.* (PA) extract, for example, has been reported to alleviate DN through the amelioration of renal tubular fibrosis and glomerulosclerosis [26]. *Ranunculus ternatus Thunb* (RTT) was shown to suppress fibrotic and inflammatory responses, thereby protecting against DN-related pathogenesis [24]. Inhibitors of SGLT2 were initially developed for their hypoglycemic properties but have also been shown to protect against reductions in estimated glomerular filtration rate (eGFR) and the onset of ESRD in clinical trials [25]. Recent evidence suggests that these SGLT2 inhibitors can protect against DN via the regulation of RAAS and the suppression of systemic oxidative stress and inflammatory activity [38].

In biclustering analyses, the diagnosis and etiology of DN were always clustered together, with both exhibiting the same dynamic changes over time in strategic diagram analyses, moving from quadrant IV from 2001 to 2010 to quadrant I from 2011 to 2021. This suggests that research output pertaining to the diagnosis and etiology of DN has not decreased over the past decade but that this research area has undergone gradual maturation. From 2001 to 2005, DN was primarily diagnosed based upon elevated levels of proteinuria [39], with many researchers having posited that the pathogenesis of DN was tied to dysregulated metabolic activity [40]. From 2006 to 2010, the eGFR served as a key diagnostic criterion for DN together with the development of proteinuria [41]. Biclustering analyses revealed that research focused on the etiological basis for DN during this time period was largely focused on detailed single-gene polymorphism-related studies, consistent with the SNA results. Indeed, several single-nucleotide polymorphisms (SNPs) were found to be related to DN susceptibility during this time frame [42, 43]. Since 2011, a growing number of studies have explored the value of blood- or urine-based biomarkers as tools for early DN diagnosis, including inflammatory chemokines, exosomes, microRNAs (miRNAs), oxidative stress-related factors, and fibrosis-related factors [5, 29, 44]. Insight into the molecular and cellular bases have also grown over the past 11 years, with research having conclusively demonstrated that immune cells, podocytes, endothelial cells, and mesangial cells all play a role in DN onset and progression [31]. Fibrosis-related factors, inflammatory chemokines, and many other mediators have also been shown to influence the pathogenesis of this condition [45].

Podocyte metabolism in DN was not a high-frequency MeSH term/subheading from 2001 to 2010, whereas it newly emerged within quadrant III of the constructed strategic diagrams from 2011 to 2021, suggesting it to be an emerging topic of research interest during this 10-year interval. Podocyte metabolism in DN similarly emerged from 2011 to 2015 when conducting SNAs, suggesting this to be a future hotspot for sustained research interest. Proteinuria is a key symptom of early-stage DN, and podocyte loss directly contributes to the development of such proteinuria, suggesting that dysregulated podocyte metabolism is a key driver of early DN pathogenesis [46]. Abnormal podocyte morphology and functionality, including the apoptotic death and epithelial-mesenchymal transdifferentiation of these cells, have also been linked to DN-associated proteinuria [47]. Recent research [48] has shown the pyroptotic death of podocytes to be an inflammatory mechanism related to DN, with the inhibition of mitochondrial damage being sufficient to inhibit this pyroptotic cell death and to thereby protect against kidney damage in murine model systems, highlighting abnormal podocyte metabolic activity as a direction for DN-focused therapeutic research interest.

The strategic diagrams generated herein highlighted DN-related signal transduction, miRNA, and mesangial cell metabolism as new emerging research directions in the period from 2016 to 2021 that were not areas of major research focus during the period from 2001 to 2015. These three topics were biclustered into Cluster 4 in quadrant III, with the positioning of this cluster further supporting the identification of these topics as emerging research directions from 2016 to 2021. Consistently, the SNA constructed for the 2016-2021 period in which emerging research directions were represented with red squares confirmed the new emergence of DN-related signal transduction, miRNA, and mesangial cell metabolism research (Figure 7(d)), while also supporting their likely status as important hotspots for future research. Several signal transduction pathways have been linked to the pathogenesis and progression of DN. For example, DN-related fibrosis can be driven by TGF-*β*/Smad signal transduction pathway activation [49]. Certain plant-derived drugs have also been shown to modulate specific signaling pathways to protect against or alleviate DN, as in the case of cardamonin, which can regulate JAK/STAT and PI3K/AKT pathways to protect rats against diabetic kidney damage [50]. Several reports have also affirmed the importance of miRNAs in the pathogenesis of DN, with certain miRNAs having been identified as valuable biomarkers that can aid in the diagnosis and prognostic evaluation of DN patients [51]. Given their role as active regulators of DN development and progression [52], many miRNAs have been confirmed to offer value as therapeutic targets in the treatment of DN [53]. Mesangial cells can modulate DN pathogenesis through the shaping of fibrotic, inflammatory, and oxidative stress activity [54]. Drugs derived from plants can ameliorate mesangial cell metabolism, thereby effectively treating DN [26]. Notably, miRNAs, signal transduction pathways, and mesangial cells are all interconnected with one another such that miRNAs can modulate key signaling pathways to modulate the metabolic activity of mesangial cells, thus affecting the development and severity of DN [55].

### 4.1. Implications

The results of the strategy diagrams and SNAs in this study are consistent with one another, thus reaffirming the rigor and objectivity of the employed bibliometric approach. Overall, these analyses highlight the research structure of the DN field and corresponding research hotspots since the beginning of the 21^st^ century, clarifying the key research direction during each of four selected periods (2001-2005, 2006-2010, 2010-2015, and 2016-2021). Software was further used to visualize these bibliometric results, thus providing clear, detailed, intuitive insight regarding the structure of this field. By clarifying the dynamic evolution of this research space and the ever-changing nature of DN-related research hotspots over the past two decades, it was also possible to predict future areas of intensive research interest associated with DN in a scientific and effective manner.

### 4.2. Limitations

There are several limitations to this analysis. For one, while PubMed is a comprehensive database that is aimed at retrieving a complete catalog of relevant scientific journal articles through multiple channels, this was the only database analyzed for the present study, potentially resulting in certain studies having been omitted from this bibliometric analysis. Second, as PubMed is an English language database, certain studies published in other languages may have been omitted. Third, this study only surveyed research pertaining to DN published within the last 21 years. Fourth, only high-frequency MeSH terms/MeSH subheadings were included in biclustering analyses, strategy diagrams, and SNAs in this study such that emerging MeSH terms/MeSH subheadings may have been overlooked by this analytical approach due to their less frequent usage, thus impacting the results of this analysis.

## 5. Conclusions

In summary, the present bibliometric analysis surveyed DN-related studies published from 2001 to 2021 in the PubMed database. Several analyses of this research space were then conducted, including time period-stratified co-word analyses, the extraction of high-frequency MeSH terms/MeSH subheadings associated with the selected time periods, biclustering analyses, and the generation of strategic diagrams and SNAs. These approaches revealed that studies of the drug-based treatment, etiology, and diagnosis of DN, along with other topics, have been hotspots of intensive DN-focused research interest from 2001 to 2021. Importantly, these results emphasize the dynamic changes in research content over the past two decades in this field, with DN-related podocyte metabolism, mesangial cell metabolism, miRNAs, and signal transduction having been identified as emerging research hotspots over the past 10 years that may thus represent critical directions for future research in the DN field.

## Figures and Tables

**Table 1:** Temporal distributions of DN-related publications in PubMed (2001–2005, 2006–2010, 2011–2015, and 2016–2021).

Period	Rank	Country	Publications, *n* (%)		Top journal	Publications, *n* (%)	
2001–2005	1	United States	1741	(42.7)	Kidney International	203	(4.9)
	2	England	678	(16.6)	Diabetes Care	197	(4.8)
	3	Germany	306	(7.5)	Nephrol Dialysis Transplant	169	(4.1)
	4	Japan	213	(5.2)	American Journal of Kidney Diseases	135	(3.3)
	5	Switzerland	187	(4.6)	Journal of the American Society of Nephrology	109	(2.7)
	6	China	111	(2.7)	Nihon Rinsho	106	(2.6)
	7	France	92	(2.3)	Diabetologia	95	(2.3)
	8	Spain	86	(2.1)	Transplantation Proceedings	94	(2.3)
	9	Netherlands	85	(2.1)	Diabetes	93	(2.3)
	10	Ireland	79	(1.9)	Diabetic Medicine	87	(2.1)
	Total		3578	(87.7)		1288	(31.4)

2006–2010	1	United States	1588	(39.6)	Nephrology Dialysis Transplantation	182	(4.5)
	2	England	775	(19.3)	Diabetes Care	143	(3.6)
	3	Germany	281	(7.0)	Kidney International	135	(3.4)
	4	Japan	182	(4.5)	Journal of the American Society of Nephrology	119	(3.0)
	5	Switzerland	145	(3.6)	Diabetes	112	(2.8)
	6	Ireland	125	(3.1)	American Journal of Physiology-Renal Physiology	107	(2.7)
	7	China	109	(2.7)	Diabetes Research and Clinical Practice	104	(2.6)
	8	Netherlands	108	(2.7)	Diabetologia	93	(2.3)
	9	Australia	73	(1.8)	Transplantation Proceedings	77	(1.9)
	10	France	70	(1.7)	Diabetic Medicine	62	(1.5)
	Total		3456	(86.0)		1134	(28.3)

2011–2015	1	United States	1793	(37.9)	PLOS ONE	146	(3.1)
	2	England	966	(20.4)	Kidney International	128	(2.7)
	3	Germany	321	(6.8)	Journal of Diabetes and Its Complications	106	(2.2)
	4	Netherlands	216	(4.6)	American Journal of Physiology-Renal Physiology	104	(2.2)
	5	Switzerland	195	(4.1)	Diabetes Care	103	(2.2)
	6	Japan	165	(3.5)	Diabetologia	91	(1.9)
	7	China	163	(3.4)	Nephrology Dialysis Transplantation	88	(1.8)
	8	Ireland	129	(2.7)	Diabetes	87	(1.8)
	9	Italy	84	(1.8)	Journal of the American Society of Nephrology	81	(1.7)
	10	Spain	81	(1.7)	Diabetic Medicine	78	(1.6)
	Total		4113	(86.9)		1012	(21.2)

2016–2021	1	United States	1992	(32.8)	Scientific Reports	170	(2.8)
	2	England	1558	(25.7)	Journal of Diabetes Research	159	(2.6)
	3	Netherlands	422	(7.0)	Journal of Diabetes and Its Complications	154	(2.5)
	4	Switzerland	343	(5.7)	Kidney International	115	(1.9)
	5	Germany	307	(5.1)	American Journal of Physiology-Renal Physiology	104	(1.7)
	6	Japan	198	(3.3)	PLOS ONE	103	(1.7)
	7	Ireland	140	(2.3)	Diabetes Care	99	(1.6)
	8	Australia	127	(2.1)	International Journal of Molecular Sciences	94	(1.5)
	9	France	119	(2.0)	Diabetes	88	(1.4)
	10	Italy	117	(1.9)	Journal of the American Society of Nephrology	87	(1.4)
	Total		5323	(87.9)		1173	(19.1)

**Table 2:** Cluster analysis of high-frequency MeSH terms/subheadings associated with DN during the 2001–2005, 2006–2010, 2011–2015, and 2016–2021 intervals.

Period	Cluster	Number of MeSH terms	Cluster analysis
2001–2005	0	33, 3, 23, 10, 44, 1, 17, 45, 15, 12, 38, 43	(1) Drug therapy of DN
	1	34, 14, 32, 26, 5, 21, 30, 25, 28, 35, 41	(1) Epidemiology of DN
			(2) Genetic polymorphism of DN
			(3) Kidney transplantation on DN
	2	39, 13, 20, 2, 47, 24, 37, 19, 29, 22, 18, 7	(1) Pathology of DN
			(2) Physiopathology of DN
			(3) Dialysis on DN
	3	46, 6, 40, 9, 36, 27, 8, 4, 16, 11, 31, 42	(1) Metabolism of DN
			(2) Diagnosis of DN
			(3) Etiology of DN

2006–2010	0	32, 22, 2, 30, 12, 1, 16, 21, 36, 28, 27	(1) Drug therapy of DN
	1	44, 25, 35, 33, 46, 42, 11, 5, 29, 31	(1) Metabolism of DN
			(2) Surgery of DN
			(3) Pathology of DN
	2	48, 10, 34, 9, 37, 18, 8, 24, 47, 17, 14, 43, 23	(1) Epidemiology of DN
			(2) Dialysis on DN
	3	38, 4, 20, 40, 26, 6, 7, 15, 41, 13, 39, 19, 3, 49, 45	(1) Single-gene polymorphism of DN
			(2) Physiopathology of DN
			(3) Diagnosis of DN
			(4) Etiology of DN

2011–2015	0	32, 31, 41, 33, 40, 1, 38, 47, 20, 12, 17, 4, 15, 42	(1) Drug therapy of DN
	1	37, 21, 6, 23, 28, 13, 10, 14, 7, 3, 49, 16	(1) Physiopathology of DN
			(2) Epidemiology of DN
			(3) Diagnosis of DN
			(4) Etiology of DN
	2	46, 8, 30, 35, 36, 22, 48, 44, 43, 18, 9	(1) Dialysis on DN
			(2) Surgery of DN
			(3) Metabolism of DN
	3	45, 24, 11, 29, 25, 19, 26, 2, 34, 27, 5, 39	(1) Pathology of DN
			(2) Podocyte metabolism in DN
			(3) Single-gene polymorphism of DN

2016–2021	0	47, 10, 28, 44, 6, 3, 9, 21, 19	(1) Epidemiology of DN
			(2) Diagnosis of DN
			(3) Etiology of DN
	1	54, 29, 39, 17, 7, 5, 16, 46, 1, 14, 31, 52, 38	(1) Drug therapy of DN
	2	58, 33, 4, 36, 25, 15, 41, 51, 13, 18, 59	(1) Single-gene polymorphism of DN
			(2) Dialysis on DN
	3	27, 11, 32, 40, 57, 43, 20, 12, 30, 50, 56, 55	(1) Physiopathology of DN
			(2) Metabolism of DN
	4	45, 42, 35, 53, 48, 24, 2, 26, 22, 34, 49, 23, 8, 37	(1) Podocyte metabolism in DN
			(2) Signal transduction of DN
			(3) MicroRNAs of DN
			(4) Mesangial-cell metabolism in DN

## Data Availability

The authors confirm that the data supporting all the results in this study are available in the article.
